# Gastrointestinal/genitourinary perforation and fistula formation with or without bevacizumab in patients with previously irradiated recurrent cervical cancer: a Korean multicenter retrospective study of the Gynecologic Oncology Research Investigators Collaboration (GORILLA) group (GORILLA-1001)

**DOI:** 10.1186/s12885-022-09695-x

**Published:** 2022-06-02

**Authors:** Woo Yeon Hwang, Suk-Joon Chang, Hee Seung Kim, Nam Kyeong Kim, Tae Hun Kim, Yeorae Kim, Tae Wook Kong, Eun Ji Lee, Soo Jin Park, Seung Hyuk Shim, Joo-Hyuk Son, Dong Hoon Suh, Eun Jung Yang

**Affiliations:** 1 Department of Obstetrics and Gynecology, Seoul National University Bundang Hospital, Seongnam, Republic of Korea; 2 Department of Obstetrics and Gynecology, Seoul National University College of Medicine, Seoul, Republic of Korea; 3 Department of Obstetrics and Gynecology, Ajou University School of Medicine, Suwon, Republic of Korea; 4 Department of Obstetrics and Gynecology, Seoul Metropolitan Government Seoul National University Boramae Medical Center, Seoul, Republic of Korea; 5 Department of Obstetrics and Gynecology, Research Institute of Medical Science, Konkuk University School of Medicine, Seoul, Republic of Korea

**Keywords:** Cervical cancer, Chemotherapy, Bevacizumab, Complication, Radiation

## Abstract

**Background:**

This study aims to evaluate the incidence of and identify risk factors for gastrointestinal (GI) and genitourinary (GU) fistula or perforation formation with or without bevacizumab in patients with recurrent cervical cancer who underwent pelvic radiation therapy (RT).

**Methods:**

Medical records of patients with recurrent cervical cancer who previously underwent pelvic RT between 2007 and 2020 were retrospectively reviewed. Clinicopathological factors were compared between groups that are stratified according to: 1) fistula/perforation (+) versus (-); and 2) bevacizumab plus conventional chemotherapy (BC) versus chemotherapy alone (C). Univariate and multivariate regression analyses were performed to identify risk factors for fistula/perforation. Overall survival (OS) was compared between the different groups.

**Results:**

Of 219 participants, fistula/perforation of any grade occurred in 36 patients (16.4%); 27 fistulas and 9 perforations. Bevacizumab was more frequently used in Bevacizumab was more frequently used ( +) group than fistula/perforation (-) group (*p* = 0.015). Multivariate analysis showed that bevacizumab administration was the only independent risk factor for fistula or perforation (HR, 3.27; 95% CI, 1.18–9.10; *P* = 0.023). F/P was observed more frequently in women receiving BC (*n* = 144) than those receiving C (*n* = 75) (20.8% vs. 8.0%; *P* = 0.019). During median follow-up of 33.7 months (1.2–185.6 months), no significant OS difference was observed between fistula/perforation ( +) vs. (-) (hazards ratio [HR], 1.78; median 84.2 months [95% CI, 59.3–109.0] vs. 129.5 months [95% CI, 114.1–144.9]; *P* = 0.065) or BC vs. C (HR, 1.03; median 119.8 months [95% CI, 97.3–142.3] vs. 115.7 months [95% CI, 96.0–135.4]; *P* = 0.928).

**Conclusions:**

This study suggests that incorporation of bevacizumab in chemotherapy regimens for treating recurrent cervical cancer in patients who underwent pelvic RT incurs considerable risk for GI/GU fistula or perforation. There were no other independent risk factors for developing GI/GU fistula or perforation in this study population.

**Supplementary Information:**

The online version contains supplementary material available at 10.1186/s12885-022-09695-x.

## Background

A significant number of patients with cervical cancer present with metastatic disease or experience recurrence after primary treatment [1]. Patients diagnosed with early stage cervical cancer may be cured using radical surgery, pelvic radiation therapy (RT), or both, while those with recurrent or persistent cervical cancer after RT have limited treatment options [2–4]. Platinum-based chemotherapy has been the mainstay of treatment for patients with recurrent disease who are not candidates for surgery or RT [5]. However, the mortality rate in patients with recurrent cervical cancer remains high. Therefore, there is a persistent need for safer and more effective therapies to prolong survival.

Angiogenesis, through a complex process involving vascular growth factors, plays a crucial role in the growth, progression, and metastasis of cervical cancer [6, 7]. Clinical evidence has been accumulating over the last decade regarding the efficacy of targeting vascular endothelial growth factor (VEGF) in cervical cancer. Bevacizumab, a recombinant humanized VEGF-neutralizing monoclonal antibody, was approved by the United States Food and Drug Administration for women with persistent, recurrent, or metastatic cervical cancer in August 2014 based on the Gynecologic Oncology Group (GOG) 240 study results [8]. GOG 240, a phase III randomized trial, showed a significant overall survival (OS) benefit conferred by the incorporation of bevacizumab in patients with recurrent, persistent, or metastatic cervical cancer [9]. While GOG 240 has resulted in a shift of standard treatment, adverse reactions such as gastrointestinal (GI) and genitourinary (GU) fistulas occurred at a higher frequency in patients treated with bevacizumab compared to patients treated with chemotherapy alone (C), and all patients who developed a fistula had a history of prior pelvic RT.

Following the GOG 240 study, the use of bevacizumab has increased for persistent, recurrent, or metastatic cervical cancer in Korea. However, there is a paucity of data regarding the fistulas or perforations involving the bowel and bladder, especially in patients with previously irradiated recurrent cervical cancer. In addition, although randomized controlled trials show significant results in terms of efficacy and safety, they do not always represent the real-world setting [10]. The purpose of this study is to evaluate the incidence rate and identify the risk factors for GI and GU fistula/perforation in patients receiving bevacizumab plus conventional chemotherapy (BC) for recurrent cervical cancer after pelvic RT in the Korean population. This study could provide evidence to guide counseling on the safety of the incorporation of bevacizumab in the treatment regimens of patients with recurrent cervical cancer who have previously undergone pelvic RT.

## Methods

### Study population

This multicenter retrospective study was conducted in accordance with the principles of the Declaration of Helsinki at five university hospitals in Korea. After approval from the institutional review boards, the medical records of patients diagnosed with recurrent cervical cancer, who had previously undergone pelvic RT between 2007 and 2020, were retrospectively reviewed. The requirement for written informed consent for the data collection was waived.

Eligible patients had been treated with external beam pelvis radiotherapy (EBRT) or concurrent chemoradiotherapy (CCRT). For RT, 3-dimentional conformal radiation therapy (3D-CRT) or intensity-modulated radiation therapy (IMRT) was performed. Patients were treated with chemotherapy either with bevacizumab for at least 1 cycle or without bevacizumab for at least 3 cycles at recurrence. Patients treated with BC as primary treatment, those treated with BC before RT, those who have received prior pelvic RT for the treatment of other malignancies, those with concomitant malignancy or a history of other malignancies being present within the past five years, and those with a lack of clinical information or loss to follow-up during treatment were excluded. Women who received chemotherapy immediately after RT were also excluded.

### Data collection

Medical records were collected, including clinicopathological data such as age, history of diabetes mellitus or hypertension, year of diagnosis, histological type, and International Federation of Gynecology and Obstetrics (FIGO) stage. We restaged the patients according to the 2018 FIGO staging system. The details of primary treatment and RT, including RT modality, application of IMRT, cumulative dose of RT, and line of RT, were collected. In addition, the total number of recurrences; whether bevacizumab was administered with chemotherapy; and adverse events, including GI and GU fistula/perforation that occurred in patients, were sorted according to the Common Terminology Criteria for Adverse Events (CTCAE) version 5.0.

### Statistical analysis

The analysis was conducted based on the occurrence of fistula/perforation and whether bevacizumab was administered, divided into fistula/perforation (+) and fistula/perforation (-) groups, and BC and C groups, respectively. Student’s *t*-test and Mann–Whitney *U* test were performed to compare continuous variables. Pearson’s chi-squared test or Fisher’s exact test was used to compare categorical variables. Univariate logistic regression analysis was performed to identify the risk factors for GI/GU fistula and perforation. Some of the risk factors in the univariate analysis were included in the multivariate logistic regression. In terms of survival outcomes, OS was calculated from the date of disease diagnosis to the date of the last follow-up or death due to any cause. The Kaplan–Meier survival curve with a log-rank test was used to compare survival outcomes. All analyses were performed using SPSS statistical software (version 21.0; SPSS Inc., Chicago, IL, USA). Statistical significance was set at *P* < 0.05.

## Results

A total of 219 patients were included in the analysis, consisting of 36 (16.4%) in the fistula/perforation (+) group and 183 (83.6%) in the fistula/perforation (-) group. The baseline patient characteristics are presented in Table 1. No differences in patient age, history of diabetes mellitus and hypertension, year of diagnosis, histologic type, 2018 FIGO stage, primary treatment, RT modality, application of IMRT, cumulative dose of RT, when patients received RT, and total number of recurrences were observed between the two groups. However, more patients in fistula/perforation (+) group had received BC than fistula/perforation (-) group (83.3% vs. 62.3%; *P* = 0.015).

**Table 1:** Clinicopathologic characteristics of the fistula/perforation (+) and (-) groups

Characteristics	All (*n* = 219)	F/P ( +)^a^ (*n* = 36)	F/P (-) (*n* = 183)	*P* value
Age, years	51.0 ± 12.6	47.8 ± 12.0	51.6 ± 12.6	0.119
DM	16 (7.3)	2 (5.6)	14 (7.7)	> 0.999
HTN	36 (16.4)	3 (8.3)	33 (18.0)	0.151
Diagnosis, year				0.258
2007–2013	66 (30.1)	8 (22.2)	58 (31.7)	
2014–2020	153 (69.9)	28 (77.8)	125 (68.3)	
Histology				0.899
SCC	144 (65.8)	24 (66.7)	120 (65.6)	
Non-SCC	75 (34.2)	12 (33.3)	63 (34.4)	
FIGO stage				0.831
I	61 (27.9)	11 (30.6)	50 (27.3)	
II	46 (21.0)	7 (19.4)	39 (21.3)	
III	89 (40.6)	14 (38.9)	75 (41.0)	
IV	23 (10.5)	4 (11.1)	19 (10.4)	
Primary treatment				0.312
OP	14 (6.4)	3 (8.3)	11 (6.0)	
OP + RT	95 (43.4)	12 (33.3)	83 (45.4)	
RT	85 (38.8)	19 (52.8)	66 (36.1)	
CTx	1 (0.5)	0 (0.0)	1 (0.5)	
Others	24 (11.0)	2 (5.6)	22 (12.0)	
RT modality				0.330
EBRT alone	23 (10.5)	2 (5.6)	21 (11.5)	
EBRT + ICR	3 (1.4)	0 (0.0)	3 (1.6)	
CCRT	118 (53.9)	24 (66.7)	94 (51.4)	
CCRT + ICR	75 (34.2)	10 (27.8)	65 (35.5)	
IMRT	85 (38.8)	17 (47.2)	68 (37.2)	0.257
RT dose				
EBRT, Gy	54.3 ± 13.7	50.9 ± 8.8	51.1 ± 6.3	0.763
ICR, Gy	23.4 ± 12.1	26.9 ± 20.9	23.0 ± 10.5	0.582
RT				0.960
Primary	188 (85.8)	31 (86.1)	157 (85.8)	
After 1^st^ recur	31 (14.2)	5 (13.9)	26 (14.2)	
Total no of recur				0.470
1	93 (42.5)	15 (41.7)	78 (42.6)	
2	75 (34.2)	10 (27.8)	65 (35.5)	
≥ 3	51 (23.3)	11 (30.6)	40 (21.9)	
Bevacizumab administration				0.015
Yes	144 (65.8)	30 (83.3)	114 (62.3)	
No	75 (34.2)	6 (16.7)	69 (37.7)	

Values are presented as mean ± standard deviation or n (%) unless otherwise indicated

*F/P* Fistula/Perforation, *DM* Diabetes Mellitus*, HTN* Hypertension, *SCC* Squamous Cell Carcinoma, *FIGO* International Federation of Gynecology and Obstetrics, *OP* Operation, *RT* Radiation Therapy, *CTx* Chemotherapy, *EBRT* External Beam Radiation Therapy, *ICR* Intracavitary Radiotherapy, *CCRT* Concurrent Chemoradiotherapy, *IMRT* Intensity-modulated Radiation Therapy

^a^ Fistula/perforation (+) includes gastrointestinal / genitourinary fistula and gastrointestinal perforation

We performed a subgroup analysis of the patients who received BC. The characteristics of the fistula/perforation (+) group (*n* = 30) and fistula/perforation (-) group (*n* = 114) of patients treated with bevacizumab are presented in Table 2. Among the variables, two were significantly different between the two groups. The fistula/perforation (+) group had fewer patients with a history of hypertension (6.7% vs. 22.8%; *P* = 0.047) and received fewer cycles of bevacizumab (5.5 ± 3.9 vs 6.7 ± 3.6; *P* = 0.028) than the fistula/perforation (+) group.

**Table 2:** Comparison of characteristics between fistula/perforation (+) and (-) groups in patients who received bevacizumab (*n* = 144)

Characteristics	F/P ( +)^a^ (*n* = 30)	F/P (-) (*n* = 114)	*P* value
Age, years	48.1 ± 12.5	53.2 ± 13.3	0.084
DM	2 (6.7)	10 (8.8)	> 0.999
HTN	2 (6.7)	26 (22.8)	0.047
Diagnosis, year			0.780
2007–2013	5 (16.7)	17 (14.9)	
2014–2020	25 (83.3)	97 (85.1)	
Histology			0.729
SCC	21 (70.0)	76 (66.7)	
Non-SCC	9 (30.0)	38 (33.3)	
FIGO stage			0.915
I	10 (33.3)	31 (27.2)	
II	5 (16.7)	23 (20.2)	
III	12 (40.0)	49 (43.0)	
IV	3 (10.0)	11 (9.6)	
Primary treatment			0.987
OP	2 (6.7)	9 (7.9)	
OP followed by adjuvant RT	12 (40.0)	43 (37.7)	
RT	15 (50.0)	59 (51.8)	
Others	1 (3.3)	3 (2.6)	
RT modality			0.123
EBRT alone	1 (3.3)	8 (7.0)	
EBRT + ICR	0 (0.0)	0 (0.0)	
CCRT	21 (70.0)	56 (49.1)	
CCRT + ICR	8 (26.7)	50 (43.9)	
IMRT	15 (50.0)	57 (50.0)	> 0.999
RT dose			
EBRT, Gy	52.9 ± 11.7	53.4 ± 12.5	0.690
ICR, Gy	23.5 ± 8.2	23.0 ± 6.4	0.400
RT			0.755
Primary	26 (86.7)	101 (88.6)	
After 1^st^ recur	4 (13.3)	13 (11.4)	
Total no of recur			0.466
1	13 (43.3)	46 (40.4)	
2	8 (26.7)	43 (37.7)	
≥ 3	9 (30.0)	25 (21.9)	
BEV administered at			0.371
1^st^ recurrence	24 (80.0)	100 (87.7)	
≥ 2^nd^ recurrence	6 (20.0)	14 (12.3)	
BEV administered cycle	5.5 ± 3.9	6.7 ± 3.6	0.028
Interval between RT and BEV administration, month	16.3 ± 23.2	18.0 ± 17.7	0.212

Values are presented as mean ± standard deviation or n (%) unless otherwise indicated

*F/P* Fistula/Perforation, *DM* Diabetes Mellitus, *HTN* Hypertension, *SCC* Squamous Cell Carcinoma, *FIGO* International Federation of Gynecology and Obstetrics, *OP* Operation, *RT* Radiation Therapy, *EBRT* External Beam Radiation Therapy, *ICR* Intracavitary Radiotherapy, *IMRT* Intensity-modulated Radiation Therapy, *BEV* Bevacizumab

^a^ Fistula/perforation (+) includes gastrointestinal / genitourinary fistula and gastrointestinal perforation

The complications of GI and GU fistula/perforation in the C and BC groups reported in this study are shown in Table 3. The overall cumulative incidence rates of fistula/perforation were 8.0% (6/75) and 20.8% (30/144) in the C group and BC group, respectively (hazards ratio [HR], 3.026; 95% confidence interval [CI], 1.199–7.640; *P* = 0.019). Although all complications of fistula/perforation in C group were severe ones with grade 3 or 4, the proportion of severe complications was still higher in BC group than C group (19.4% vs. 8.0%; *P* = 0.029).

**Table 3:** Occurrence of fistula and perforation according to bevacizumab administration

	C (*n* = 75)	BC (*n* = 144)	RR (95% CI)	*P* value
GI fistula				
Grade 2	0	1 (0.5)	NA	> 0.999
Grade 3	2 (0.9)	5 (2.3)	1.313 (0.249–6.934)	0.748
Grade 4	0	3 (1.4)	NA	0.999
GU fistula				
Grade 2	0	1 (0.5)	NA	> 0.999
Grade 3	1 (0.5)	5 (2.3)	2.662 (0.305–23.208)	0.376
Grade 4	0	1 (0.5)	NA	> 0.999
GI fistula & GU fistula				
Grade 3	0	7 (3.2)	NA	0.999
Grade 4	0	1 (0.5)	NA	> 0.999
GI perforation				
Grade 3	2 (0.9)	4 (1.8)	1.043 (0.187–5.829)	0.962
Grade 4	1 (0.5)	2 (0.9)	1.042 (0.093–11.684)	0.973
Total				
-	6 (8.0)	30 (20.8)	3.026 (1.199–7.640)	0.019

Values are presented as n (%) unless otherwise indicated

*C *Chemotherapyalone*, BC *Bevacizumab plus Conventional chemotherapy*, RR* Risk Ratio, *CI* Confidence Interval, *NA* Not Available, *GI* Gastrointestinal, *GU* Genitourinary

The multivariate analysis in Table 4 revealed that administration of bevacizumab appeared to be the only independent risk factor for occurrence of fistula/perforation (HR, 3.273; 95% CI, 1.177–9.096; *P* = 0.023).

**Table 4:** Univariate and multivariate analyses of the risk factors for GI/GU fistula and perforation

Variables	Univariate			Multivariate		
	HR	95% CI	*P* value	HR	95% CI	*P* value
Age						
< 50	1			1		
≥ 50	0.577	0.278–1.197	0.140	0.666	0.307–1.445	0.304
DM						
No	1					
Yes	0.710	0.154–3.269	0.710			
HTN						
No	1			1		
Yes	0.413	0.120–1.429	0.163	0.426	0.116–1.566	0.199
Diagnosis, year						
2007–2013	1			1		
2014–2020	1.624	0.697–3.781	0.261	0.885	0.317–2.469	0.815
Histology						
Non-SCC	1					
SCC	1.050	0.492–2.239	0.899			
FIGO stage						
I-II	1					
III-IV	0.947	0.463–1.935	0.881			
IMRT						
No	1			1		
Yes	1.513	0.737–3.108	0.259	1.136	0.498–2.592	0.761
RT						
Primary	1					
After 1^st^ recur	0.974	0.347–2.733	0.960			
Bevacizumab administration						
No	1			1		
Yes	3.026	1.199–7.640	0.019	3.273	1.177–9.096	0.023

*HR* Hazard Ratio, *CI* Confidence Interval, *DM* Diabetes Mellitus, *HTN* Hypertension, *SCC* Squamous Cell Carcinoma, *FIGO* International Federation of Gynecology and Obstetrics, *IMRT* Intensity-modulated radiation therapy, *RT* Radiation Therapy

In addition, we compared the C and BC groups. Of 219 patients, 75 (34.2%) received C, and 144 (65.8%) received BC (Supplementary Table S1). Compared with group C, group BC had a significantly higher proportion of patients who were diagnosed in later period between 2014 and 2020 (41.3% vs. 84.7%; *P* < 0.001). Regarding RT modality, patients who received RT only for primary treatment (14.7% vs. 51.4%; *P* < 0.001), CCRT with ICR (22.7% vs. 40.3%; *P* < 0.001) and IMRT (17.3% vs. 50.0%; *P* < 0.001) were more frequent in group BC than group C. Patients who were treated by EBRT alone, however, were more frequent in group C than group BC (18.7% vs. 6.3%; *P* < 0.001).Multivariate analyses of the risk factors for administration of bevacizumab showed that year of diagnosis (HR, 9.704; 95% CI, 4.171–22.579; *P* < 0.001) and use of IMRT (HR, 2.192; 95% CI, 1.009–4.762; *P* = 0.047) were independent risk factors for administration of bevacizumab (Supplementary Table S2). Since the GOG 240 study was published in 2014, we performed another sub-group analysis of risk factors for fistula/perforation only in the patients after 2014 (Supplementary Table S3). The results showed that there were no significant risk factors related to fistula/perforation.

**Fig. 1:**
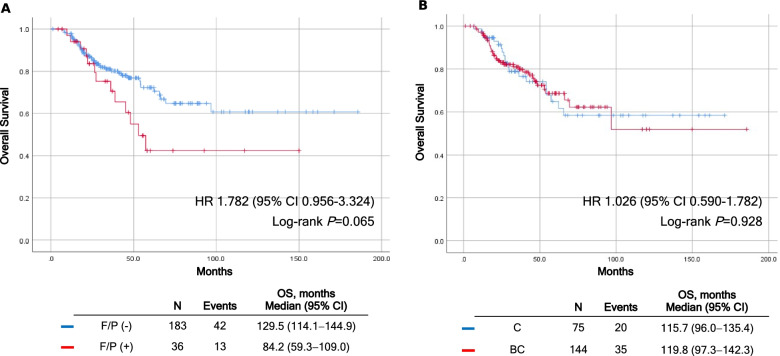
Kaplan–Meier curves of Overall survival according to **A** the occurrence of fistula/perforation and **B** the use of bevacizumab

During a median length of follow-up of 33.7 months (range, 1.2–185.6 months), 55 patients (25.1%) expired, and no significant OS difference was observed between the fistula/perforation (+) group vs. fistula/perforation (-) group (HR, 1.782; median, 129.5 months [95% CI, 114.1–144.9] vs. 84.2 months [95% CI, 59.3–109.0]; *P* = 0.065) or between C vs. BC group (HR, 1.026; median, 115.7 months [95% CI, 96.0–135.4] vs. 119.8 months [95% CI, 97.3–142.3]; *P* = 0.928) (Fig. 1).

## Discussion

Platinum-based chemotherapy with bevacizumab is one of the most promising treatment options for recurrent or persistent cervical cancer [5, 8]. However, our study results suggest that the incorporation of bevacizumab in the chemotherapy regimen for the treatment of recurrent cervical cancer in patients with a previous history of pelvic RT incurs considerable risk for GI and/or GU fistula or perforation complications. We failed to identify other independent risk factors for developing GI/GU fistula or perforation in this study population. GI and/or GU fistula or perforation may influence the survival outcomes, as well as quality of life.

Differences were found between our study and the GOG 240 study. Our results showed a notably higher rate of fistula/perforation in both groups compared to the GOG 240 study (20.8% vs. 8.0%). The GOG 240 study reported an incidence of severe fistula/perforation of 5.9% (13/220) in BC group and 0.5% (1/220) in C group [9]. All patients with fistulas had previous received RT, even if it was not indicated which modality of RT was applied. There was another study that reported an increase in fistula formation up to 22% in the real-world population [11]. In terms of OS, in our study, BC group did not show a significant improvement compared to C group, while the GOG 240 study showed a significant improvement in OS. However, the GOG 240 data on two distinct chemotherapy doublets with bevacizumab showed different results. When compared with the cisplatin plus paclitaxel chemotherapy, patients who received additional bevacizumab showed a significant OS improvement (HR 0.73 [95% CI 0.54–0.99]; *P* = 0.04), while topotecan plus paclitaxel alone and topotecan plus paclitaxel plus bevacizumab did not show a significant difference in OS [9]. Therefore, the lack of difference in OS in our study may be due to the different components of chemotherapy regimens. In Korea, weekly cisplatin and cisplatin plus paclitaxel are the most commonly-used regimens for CCRT and systemic therapy, respectively. Carboplatin plus paclitaxel, paclitaxel only and cisplatin plus topotecan are also used for some patients [12].

Our results have several interesting perspectives. IMRT delivers higher radiation doses focused on the tumor while minimizing the dose delivered to normal structures [13]. Contrary to our expectations, our study showed that IMRT does not reduce GI or GU complications neither in the overall sample of patients nor in the subgroup treated with bevacizumab. A previous meta-analysis showed results distinct from those of our study. The study compared the clinical outcomes and toxicities of IMRT with 3D-CRT or conventional two-dimensional radiotherapy (2D-RT) for definitive treatment of cervical cancer showing that although IMRT was not superior to 3D-CRT or 2D-RT in OS but it reduced acute GI and GU toxicities, as well as chronic GU toxicity [14]. However, IMRT is still associated with significant rectal and cystic toxicity. Wu et al. [15] reported higher GI toxicities in IMRT group than conventional RT group, which was consistent with our study results. Wu et al. described that there were unavoidable hot spots with uncertain high dose to rectum and bladder which may increase complications by reviewing the IMRT planning [15]. Our findings may also have been affected by high doses delivered to rectum and bladder when IMRT was administered.

In the BC group, more patients were diagnosed after 2014, and more patients were treated with IMRT (Supplement Table S1). This is thought to be related to the time when IMRT and bevacizumab were introduced to the treatment regimens for patients with gynecological cancer in Korea. The implementation of IMRT is related to the policies of the national health insurance in Korea. IMRT was first introduced in 2001 in Korea, and since July 2015, insurance coverage of this procedure was expanded to include most cancers [16]. In addition, the United States Food and Drug Administration approved bevacizumab in combination with first-line chemotherapy for patients with persistent, recurrent, or metastatic cervical cancer in 2014 based on GOG 240 [17]. Therefore, the year of diagnosis of cervical cancer and application of IMRT may have been confounded by the health insurance, the year of publication and approval.

Our study is unique compared to previous studies that addressed bevacizumab complications due the uniqueness of our study population. We evaluated fistula/perforation caused by bevacizumab in patients with previously irradiated recurrent cervical cancer. In addition, being a multicenter study with data collected from five large university hospitals in Korea makes our study results more likely to be generalized to the Korean population. However, our study had several limitations. First, there may be potential bias due to the retrospective nature of our study. Second, the insurance system and unique healthcare environment in Korea may have influenced our results. Third, we did not collect every single data on the treatment regimen for all treatment steps for CCRT or systemic chemotherapy. The difference in regimens may be a risk factor for complications or may influence OS, as mentioned above. Finally, data on the performance status of patients could not be obtained, as we collected the data from medical records.

In conclusion, our study results suggest that the incorporation of bevacizumab in chemotherapy regimens for the treatment of recurrent cervical cancer in patients with a previous history of pelvic RT incurs considerable risk of GI and GU fistula or perforation, which might compromise the survival outcome, as well as quality of life. Careful weighing of the risks and benefits of incorporating bevacizumab is needed in the treatment of patients with previously irradiated recurrent cervical cancer, considering that effective prevention of fistula/perforation complications is difficult because of the lack of other identifiable risk factors.

## Supplementary Information

**Additional file 1:** **Supplement Table S1. **Comparison of characteristics according to the use of bevacizumab. **Supplement Table S2.** Univariate and multivariate analyses of the risk factors for GI/GU fistula and perforation associated with bevacizumab use. **Supplement Table S3.** Univariate and multivariate analyses of the risk factors for GI/GU fistula and perforation associated with bevacizumab use after 2014. 

## Data Availability

The datasets used and/or analyzed during the current study are available from the corresponding author on reasonable request.

## Abbreviations

GI: Gastrointestinal
GU: Genitourinary
BC: Bevacizumab plus conventional chemotherapy
RT: Radiation therapy
F/P: Fistula/Perforation
C: Chemotherapy alone
OS: Overall survival
HR: Hazards ratio
CI: Confidence interval
VEGF: Vascular endothelial growth factor
GOG: Gynecologic Oncology Group
EBRT: External beam pelvis radiotherapy
CCRT: Concurrent chemoradiotherapy
3D-CRT: 3-Dimentional conformal radiation therapy
IMRT: Intensity-modulated radiation therapy
DM: Diabetes mellitus
FIGO: International Federation of Gynecology and Obstetrics
CTCAE: Common Terminology Criteria for Adverse Events
