# Supplementary material for: Gastrointestinal/genitourinary perforation and fistula formation with or without bevacizumab in patients with previously irradiated recurrent cervical cancer: a Korean multicenter retrospective study of the Gynecologic Oncology Research Investigators Collaboration (GORILLA) group (GORILLA-1001)
Source: BMC Cancer. 2022 Jun 2;22:603. doi: 10.1186/s12885-022-09695-x (PMC9161567; doi:10.1186/s12885-022-09695-x)
Supplement: Supplementary file 1 — Additional file 1: Supplement Table S1. Comparison of characteristics according to the use of bevacizumab. Supplement Table S2. Univariate and multivariate analyses of the risk factors for GI/GU fistula and perforation associated with bevacizumab use. Supplement Table S3. Univariate and multivariate analyses of the risk factors for GI/GU fistula and perforation associated with bevacizumab use after 2014. [file 12885_2022_9695_MOESM1_ESM.docx]

| **Supplement Table S1.** Comparison of characteristics according to the use of bevacizumab | | | |
| --- | --- | --- | --- |
| Characteristics | C  (n=75) | BC  (n=144) | *P* value |
| Age, years | 48.7 ± 11.0 | 52.2 ± 13.2 | 0.092 |
| DM | 4 (5.3) | 12 (8.3) | 0.418 |
| HTN | 8 (10.7) | 28 (19.4) | 0.096 |
| Diagnosis, year |  |  | < 0.001 |
| 2007-2013 | 44 (58.7) | 22 (15.3) |  |
| 2014-2020 | 31 (41.3) | 122 (84.7) |  |
| Histology |  |  | 0.899 |
| SCC | 120 (65.6) | 24 (66.7) |  |
| Non-SCC | 63 (34.4) | 12 (33.3) |  |
| FIGO stage |  |  | 0.925 |
| I | 20 (26.7) | 41 (28.5) |  |
| II | 18 (24.0) | 28 (19.4) |  |
| III | 28 (37.3) | 61 (42.4) |  |
| IV | 9 (12.0) | 14 (9.7) |  |
| Primary treatment |  |  | < 0.001 |
| OP | 3 (4.0) | 11 (7.6) |  |
| OP followed by adjuvant RT | 40 (53.3) | 55 (38.2) |  |
| RT | 11 (14.7) | 74 (51.4) |  |
| CTx | 1 (1.3) | 0 (0.0) |  |
| Others | 20 (26.7) | 4 (2.8) |  |
| RT modality |  |  | < 0.001 |
| EBRT alone | 14 (18.7) | 9 (6.3) |  |
| EBRT+ICR | 3 (4.0) | 0 (0.0) |  |
| CCRT | 41 (54.7) | 77 (53.5) |  |
| CCRT+ICR | 17 (22.7) | 58 (40.3) |  |
| IMRT | 13 (17.3) | 72 (50.0) | <0.001 |
| RT dose |  |  |  |
| EBRT, Gy | 56.0 ± 15.9 | 53.3 ± 12.3 | 0.708 |
| ICR, Gy | 24.3 ± 21.4 | 23.1 ± 6.6 | 0.281 |
| RT |  |  | 0.960 |
| Primary | 157 (85.8) | 31 (86.1) |  |
| After 1^st^ recur | 26 (14.2) | 5 (13.9) |  |
| Total number of recur |  |  | 0.503 |
| 1 | 78 (42.6) | 15 (41.7) |  |
| 2 | 65 (35.5) | 10 (27.8) |  |
| ≥3 | 40 (21.9) | 11 (30.6) |  |
| Values are presented as mean ± standard deviation or n (%) unless otherwise indicated.  C, chemotherapy alone; BC, bevacizumab plus conventional chemotherapy; DM, diabetes mellitus; HTN, hypertension; SCC, squamous cell carcinoma; FIGO, International Federation of Gynecology and Obstetrics; OP, operation; RT, radiation therapy; CTx, chemotherapy; EBRT, external beam radiation therapy; ICR, intracavitary radiotherapy; CCRT, concurrent chemoradiotherapy; IMRT, Intensity-modulated radiation therapy | | | |

**Supplement Table S2.** Univariate and multivariate analyses of the risk factors for GI/GU fistula and perforation associated with bevacizumab use

| Variables | Univariate | | | Multivariate | | |
| --- | --- | --- | --- | --- | --- | --- |
|  | HR | 95% CI | *P* value | HR | 95% CI | *P* value |
| Age |  |  |  |  |  |  |
| <50 | 1 |  |  |  |  |  |
| ≥50 | 1.056 | 0.604-1.845 | 0.848 |  |  |  |
| DM |  |  |  |  |  |  |
| No | 1 |  |  |  |  |  |
| Yes | 1.614 | 0.502-5.188 | 0.422 |  |  |  |
| HTN |  |  |  |  |  |  |
| No | 1 |  |  |  |  |  |
| Yes | 2.022 | 0.872-4.689 | 0.101 |  |  |  |
| Diagnosis, year |  |  |  |  |  |  |
| 2007-2013 | 1 |  |  | 1 |  |  |
| 2014-2020 | 7.871 | 4.125-15.017 | < 0.001 | 9.704 | 4.171-22.579 | < 0.001 |
| Histology |  |  |  |  |  |  |
| Non-SCC | 1 |  |  |  |  |  |
| SCC | 1.230 | 0.686-2.203 | 0.488 |  |  |  |
| FIGO stage |  |  |  |  |  |  |
| I-II | 1 |  |  |  |  |  |
| III-IV | 1.116 | 0.639-1.951 | 0.699 |  |  |  |
| IMRT |  |  |  |  |  |  |
| No | 1 |  |  | 1 |  |  |
| Yes | 4.769 | 2.413-9.426 | < 0.001 | 2.192 | 1.009-4.762 | 0.047 |
| RT |  |  |  |  |  |  |
| Primary | 1 |  |  |  |  |  |
| After 1^st^ recur | 0.583 | 0.270-1.260 | 0.170 |  |  |  |

HR, hazard ratio; CI, confidence interval; DM, diabetes mellitus; HTN, hypertension; SCC, squamous cell carcinoma; FIGO, International Federation of Gynecology and Obstetrics; IMRT, Intensity-modulated radiation therapy; RT, radiation therapy

**Supplement Table S3.** Univariate and multivariate analyses of the risk factors for GI/GU fistula and perforation associated with bevacizumab use after 2014

| Variables | Univariate | | | Multivariate | | |
| --- | --- | --- | --- | --- | --- | --- |
|  | HR | 95% CI | *P* value | HR | 95% CI | *P* value |
| Age |  |  |  |  |  |  |
| <50 | 1 |  |  | 1 |  |  |
| ≥50 | 0.507 | 0.204-1.259 | 0.143 | 0.786 | 0.300-2.062 | 0.625 |
| DM |  |  |  |  |  |  |
| No | 1 |  |  |  |  |  |
| Yes | 0.850 | 0.172-4.209 | 0.842 |  |  |  |
| HTN |  |  |  |  |  |  |
| No | 1 |  |  | 1 |  |  |
| Yes | 0.142 | 0.018-1.110 | 0.142 | 0.155 | 0.018-1.299 | 0.086 |
| Histology |  |  |  |  |  |  |
| Non-SCC | 1 |  |  | 1 |  |  |
| SCC | 1.633 | 0.595-4.480 | 0.341 | 1.601 | 0.569-4.506 | 0.373 |
| FIGO stage |  |  |  |  |  |  |
| I-II | 1 |  |  |  |  |  |
| III-IV | 0.972 | 0.401-2.357 | 0.950 |  |  |  |
| IMRT |  |  |  |  |  |  |
| No | 1 |  |  |  |  |  |
| Yes | 0.932 | 0.384-2.261 | 0.876 |  |  |  |
| RT |  |  |  |  |  |  |
| Primary | 1 |  |  | 1 |  |  |
| After 1^st^ recur | 1.517 | 0.372-6.192 | 0.561 | 1.746 | 0.393-7.750 | 0.464 |

HR, hazard ratio; CI, confidence interval; DM, diabetes mellitus; HTN, hypertension; SCC, squamous cell carcinoma; FIGO, International Federation of Gynecology and Obstetrics; IMRT, Intensity-modulated radiation therapy; RT, radiation therapy
